# Digital-Free Tourism Holiday as a New Approach for Tourism Well-Being: Tourists’ Attributional Approach

**DOI:** 10.3390/ijerph19105974

**Published:** 2022-05-14

**Authors:** Thowayeb H. Hassan, Amany E. Salem, Mahmoud I. Saleh

**Affiliations:** 1 Social Studies Department, College of Arts, King Faisal University, Al Ahsa 400, Saudi Arabia; asalem@kfu.edu.sa; 2 Tourism Studies Department, Faculty of Tourism and Hotel Management, Helwan University, Cairo 12612, Egypt; mahmoudibraheam580@gmail.com; 3 Marketing Department, Graduate School of Management, Saint Petersburg State University, 199004 Saint Petersburg, Russia

**Keywords:** digital-free tourism, tourism digitalization, digital detox, tourist well-being, millennials, tourist health, locus of control, tourism technology

## Abstract

Digital-free tourism (DFT) has recently attracted tourism service providers’ attention for its benefits in terms of enhancing tourists’ experiences and well-being at destinations. DFT refers to tourists who are likely to voluntarily avoid digital devices and the Internet on holiday, or travel to destinations without network signals. DFT has advantages for tourists in increasing well-being, mental health, and social networking during their journeys. DFT also has a benefit for tourism marketers in that they can consider it as a new tourism approach. However, there is a lack of studies into tourists’ locus of control (LOC) while experiencing DFT holidays. LOC refers to how individuals assign the responsibility of event outcomes—whether they assign it to themselves (internal LOC) or they say it is beyond their control (external LOC). Therefore, the current study contributes to investigating tourists’ LOC impacts while experiencing DFT holidays. The study relies on semi-structured interviews with millennial tourists who have experienced DFT holidays. The study findings reveal that millennial tourists with an internal LOC (vs. external) are more likely to perceive the DFT advantages (vs. obstacles) during and after the DFT holidays. However, millennial tourists with external LOC incrementally change their attitudes and perceive the DFT holiday benefits through their self-efficacy enhancement. The findings propose managerial strategies for developing effective DFT holidays for millennial tourists regarding their LOC.

## 1. Introduction

The number of tourists has significantly increased because individuals have more holidays with more disposable income [1]. The increasing number of tourists helps increase revenues for tourism destinations and provides thousands of jobs [1]. According to UNWTO [1], the number of tourists increased to 1.4 billion in 2018 with USD 1.7 trillion. It is also expected to reach 1.8 billion by 2030 because of rapid digital transformations [2]. Digital transformation and technology play a crucial role in forming consumer intentions, attitudes, purchasing behavior, and satisfaction, especially in the tourism industry [3]. Moreover, they enhance service providers’ marketing campaigns to attract consumers [4]. Thus, the increasing number of tourists has led many tourism service providers to use digital transformation advantages to attract the highest number of tourists throughout their marketing campaigns. Digital marketing allows tourism service providers to customize marketing campaigns to sell services and directly present information to tourists through websites [5].

So, tourists utilize digital tourism platforms due to their benefits in terms of booking and comparing different alternatives [3]. Digitalization in the tourism industry drives tourists to represent themselves as encountering actors negotiating and seeking better experiences [6]. Thus, tourism service providers utilize many digital tools such as mobile applications, websites, and social media to enhance consumer service, attracting new consumers to increase revenues [3]. Tourists use all these digital tools, mobile applications, and social media to improve their experience during holidays [4].

The importance of digital transformation for both tourism service providers and tourists is mainly related to seeking alternative information [7], building brand reputations [3], and enhancing tourists’ experiences at destinations [8]. However, digital technologies and/or social media negatively impact tourists’ well-being and mental health. For instance, the “e-lineation” concept has been proposed to show tourists’ negative life experiences, including superficiality, meaninglessness, dropping social norms, and self-isolation during holidays [9]. Thus, tourists find motivation to unplug from social media and digital technology during holidays because of the destructive feelings and dark traits associated with using these technologies, such as detachment from social and physical surroundings, diminished recovery, and lack of wellness balance during holidays [10].

Therefore, tourism scholars have developed a new phenomenon that describes the tendency to get rid of technologies during holidays, namely “digital-free tourism” (DFT), which refers to minimal access to, and the absence of, communication technologies and information during vacations [11]. DFT influences tourists to improve their health and personal growth by driving them to get off-line [10]. Tourism researchers have found that some tourists do not take their smartphones with them during their journeys [9] to avoid dark psychological traits when using mobile phones and any digital technologies during holidays [4]. This tendency is called “digital detox”; it introduces the idea of avoiding any virtual world on holidays by decreasing people’s habitual usage of social media and electronic devices [11].

Tourism destination managers have found that tourists’ tendencies to experience DFT holidays bring more revenues to tourism destinations that encourage this type of experience [4,9,10]. However, there is a prominent gap in studies of tourists’ self-control—namely, their locus of control (internal vs. external.) while encountering these DFT holidays. Locus of control (LOC) theory refers to the extent to which people believe that they can control events’ outcomes in their daily lives [12]. Individuals could have an internal LOC, or an external LOC. Internal LOC refers to the people who assign the responsibility of events’ outcomes to their ability to control them. In contrast, an external LOC refers to the people who assign the responsibility of events’ consequences to causes beyond their control or external factors such as luck and fate [13]. Individuals’ locus of control could influence tourists to make different decisions [12,14] because it is a consistent predictor of a tourist’s attitude, emotion, and tendencies to adopt technologies [15].

Therefore, it is crucial to pay more attention to studying tourists’ LOCs during experiencing digital-free tourism holidays. By employing the locus of control theory to understand tourists’ DFT holidays, tourism managers will find the potentials and/or the obstacles that help improve DFT experiences. Therefore, the current study examines the following question “how do tourists perceive DFT holidays advantages or disadvantages regarding their LOC (internal and external)?”.

## 2. Literature Review

### 2.1. Digital Significance for Tourists during Holidays

The Internet and globalization are two important impulses behind the importance of individual uses of digital technologies [5]. Within this context, digital technologies advocates claim that such technologies boosts service providers and consumers’ collaboration, communication and sharing, and improves information gathering [16]. The digital environment offers a wide range of data covering ratings, customer reviews, tags, blogs, social interactions, and consumers’ responses to marketing activities. These data are beneficial both for service providers in terms of understanding online consumer attitudes to develop professional marketing strategies and for consumers in terms of measuring the effectiveness of services features and choosing between competitors [17]. That is why consumers could find more alternatives in the online environment than offline customers when collecting information [16].

In the tourism context, during holidays, tourists are likely to perceive information about services online more than offline because price sensitivity is lower online than offline, and brand names and brand equity have a more substantial influence online than offline [18]. Existing literature reveals that digital experiences not only influence tourists’ mental processes and enhance online purchasing behaviors and decision making [5,6,9,19], but also help tourists during their holidays to check for alternative solutions to any problem and share their experiences with their friends [19,20]. Tourists assume that the tourism service providers’ existence (vs. not) on digital platforms is in indication of its existence (vs. absence) in the tourism market [9]. This is especially the case for millennial tourists. Millennial tourists are technologically savvy and have the ability to check the Internet and use social media for a long time [21]. According to Zhuang et al. [22], millennial tourists tend to use digital technologies more than other tourists during holidays; moreover, they are more likely to be affected by technological and digital tourism campaigns.

A study by Cuomo et al. [6] found that tourists who are enthusiastic about visiting destinations are more likely to be disappointed if they find a lack of digital presence in these destinations on social media, because it helps tourists to enhance their experiences in tourist destinations [3]. A recent study by Hassan et al. [23] found that travelers’ engagement in technology utilization during holidays is one of the more mainstream activities in tourism destinations. They also emphasized that digital platforms play a dominant role in facilitating tourists’ trip itineraries. A digital presence in holidays plays a dominant role as an information platform that can be used to attain an overview of current issues and to clarify the connections between tourism service providers and tourists to build up the destination’s image [11]. For instance, search engine queries, Google Trends, and extensive social data provide more profound insights that help tourists to enhance their experiences during holidays [20,24].

However, many studies have shed light on the dark traits associated with using digital technologies during holidays with their adverse impacts on tourists’ physical health, especially psychological well-being [11]. A large addiction to digital technology causes fear of missing out (FoMO), caused by smartphone addiction and nomophobia (the panic of not using a mobile phone) [4]. These fears and terrors lead to several mental health issues (e.g., low self-esteem, anxiety, depression, and technostress) [21]. This encourages tourists to voluntarily avoid digital technology usage during holidays, which researchers call digital-free tourism. The next section will synthesize the new tendencies to apply digital-free tourism and digital detox in the tourism context.

### 2.2. Digital-Free Tourism (DFT) and Tourists’ Locus of Control (LOC)

To treat the dark traits associated with using digital technologies at holiday destinations (e.g., smartphone addiction and nomophobia, anxiety, and depression), tourism practitioners make practical efforts to attract tourists for digital-free holidays. Digital-free tourism (DFT) refers to holidays with a lack of electronic devices or the Internet [9]. DFT could also be defined as an intentional experience by tourists, where they switch off any digital technologies during holidays [10]. This means that tourists switch off their electronic devices and/or disconnect Internet services during vacations [19,20]. For instance, “digital downtime” by the Scottish tourism industry, and “Blackhole” resorts in the United Kingdom and North America, “digital detoxing” in the Maldives where tourists disconnect from the Internet and are forbidden to use any digital technology during their holidays to treat the Internet addiction and manage tourist stress [11]. So, tourists are recommended to avoid any digital technologies to avoid obstacles when using digital technology in the tourism industry [3].

The reasons that lead tourists to respond to these calls for DFT and to dislike the “digital leash” are (a) social media addiction-prone applications such as Facebook, Twitter, and Instagram, because these are barriers to enjoying the present moments during vacations; (b) social media and technologies have a harmful influence on interpersonal relations because social media platforms hinder interactions amongst travel companions on journeys; (c) the pressure provoked by forcing them to “show and live” their experiences for others by comparing themselves with others to avoid ignoring the external “ever-present expectation” in this digital era [11]. Disliking the “digital leash” encourages tourists to benefit from the privileges of digital detox during holidays, which are creativity, curiosity, open-mindedness, love of learning, perspective, bravery, persistence, integrity, social intelligence, forgiveness and mercy, humility/modesty, prudence, self-regulation, gratitude, and spirituality [19,20]. All these character strengths before, during, and after the journey could exist because tourists do not use any digital technology [3,4,9].

Though digital detox during holidays is important, not all tourists can withstand digital withdrawal symptoms. Some tourists cannot cut off the connection because they have particular commitments, so they cannot be unavailable, or they are afraid of getting lost [4]. For instance, tourists who cannot manage their work issues may depend on digital devices to finish work-related tasks while on holiday [10]. Moreover, suffering from withdrawal symptoms during vacations differs based on the destination types. For example, tourists may experience higher levels of anxiety and frustration in urban destinations compared to rural and natural destinations due to the need for instant information access, navigation, and word-of-mouth recommendation to feel in control of their holidays [4]. Tourists who also travel in a group or as couples are less likely to have anxiety when disconnecting than solo tourists because they feel they are in control of the trip itinerary [4]. 

Given that tourists’ feelings of control influence them in terms of connecting with and/or disconnecting from digital technologies [10] and that there is a lack of studies that highlight feelings of control of tourists experiencing DFT holidays, the next part of this paper will shed light on tourists’ locus of control as a prominent theory to understand individuals’ control about surrounding events [12] to determine tourists’ tendencies and abilities to engage in DFT holidays.

Locus of control refers to individuals’ different characteristics in terms of the extent to which individuals ascribe their ability to achieve success (or failure) to their daily life events [12]. Individuals with internal LOC (“internals”) believe that their attitude and abilities can generate desired outcomes. These “internals” have a high probability of being determined, confident, and believing that they can control their daily events and fate [25]. Moreover, they have a greater probability of considering that perceived “earned” outcomes result from their power to achieve these outcomes [26]. Conversely, individuals with external LOC (“externals”) believe that causes outside themselves generate their outcomes [27]. These “externals” are more likely to assume that they are victims of external powers, environments, and circumstances they encounter [26].

Researchers in consumer psychology have clarified that LOC is one of three attribution theory dimensions—stability, controllability, and locus [28]—even though attribution theory describes how individuals explain the causes of outcomes (e.g., failures or successes) in their lives in a similar way to LOC theory [29,30]. However, attribution descriptions are usually posted temporarily and occur after a success or failure experience. By contrast, locus of control is essentially forward-looking, including insight into one’s ability to control the explained outcome. Individuals may feel that their events are caused by factors beyond their control (situational attribution); yet, they believe that they can handle it (internal LOC) by taking remedies for these events [27]. Moreover, psychological studies have demonstrated that LOC is a component of self-control. This indicates that a lower level of non-cognitive abilities may intensify problems about an individual’s self-control, especially externals more than internals [14].

Thus, when individuals have external LOC, they are more likely to be influenced by external causes and to be unable to control surrounding events well because they assign responsibility for the events to external causes [12]. In contrast, individuals with internal LOC are more likely to control surrounding events by assigning responsibility for the events to internal causes [15]. Internal LOC (vs. external) is more likely to be associated with better social experiences, better health, and better economic outcomes. For instance, individuals with internal (vs. external) LOC are more likely to cope with contradictory life shocks, have higher saving and satisfaction levels, adopt productivity-enhancing technologies, and seek better mental health [13]. Regarding tourist satisfaction, there is an inevitable argument that tourist satisfaction and well-being are interconnected. In a contemporary model suggested by Lin et al. [31], they found that, for the most part, tourists’ indicators for their well-being are actively shaped by consistency regarding their affective satisfaction at gastronomic destinations, especially concerning high-quality services. Thus, when we highlighted the LOC part, tourists who feel in control of their journeys would increase their level of satisfaction [12]; therefore, they will have a high probability of well-being with an increase of control sense [31].

From the tourist gender perspective, there is a distinction in the level of LOC between males and females. For instance, females are, compared to males, usually more likely to revolve around attributing most adverse events that require efforts (e.g., DFT) to external causes with an external LOC to avoid low self-control [32] and avoid self-blame [33]. Thus, females are likely to ask more questions to get more information that may raise their confidence during the holidays [33] because females experience different levels of LOC in several situations compared to males [34]. According to Saleh [35], female tourists attempt to avoid low self-confidence during holidays by collecting more information to increase their control during tourism service encounters. Thus, while DFT holiday tourists have to avoid using the Internet, they may encounter difficulties with Internet leverage in collecting data. Consequently, we argue that female tourists have different perceptions of DFT holidays than males regarding their LOC.

**Figure 1:**
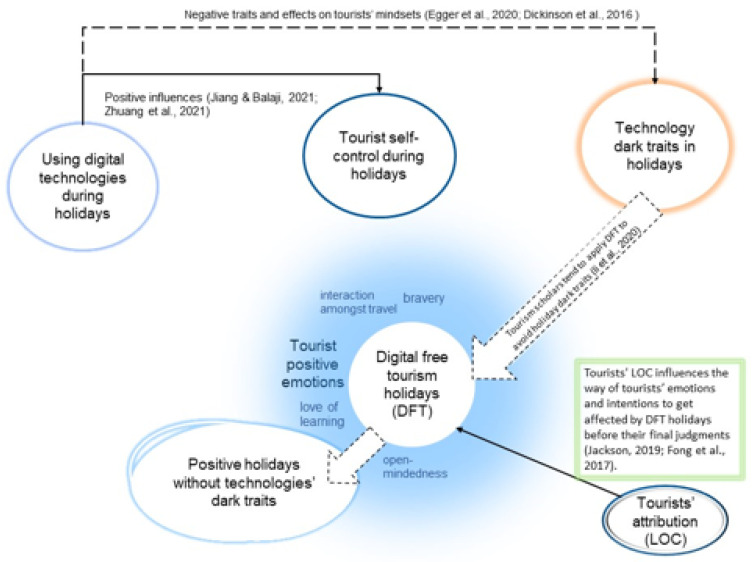
The interdependence between DFT, LOC, and tourists’ behavioral outcomes.

In this vein, the current study proposes that tourists do not take a digital-free tourism approach because of DFT advantages per se. However, the locus of control and attribution toward such an approach plays a crucial role when engaging in such holidays (Figure 1). Therefore, the current study investigates how tourists with different LOC (internal vs. external) respond to digital-free tourism holidays.

Figure 1 explains that it is needless to highlight that tourist experiences and technology usage are related [36]. Tourists aim to use digital technologies during holidays to facilitate their holiday items and collect mode data, avoiding risks during holidays [37]. Tourists also use digital platforms to share their trip photos with their peers and check news updates, leading to more holiday enjoyment [9]. Notwithstanding that digital technologies during holidays have their privileges, they still have their dark traits [10,20]. For instance, they hinder interaction in travel, increase comparisons with others, and decrease open-mindedness, love of learning, perspective, and bravery. These dark traits drive destination managers to find an alternative approach to addressing these dark trait issues, introducing a digital-free tourism approach. As mentioned earlier, tourists tend to control their journey using digital technologies. The motivation for using technology reflects the fact that tourists want to have high engagement in holiday activities [10]. Tourists’ motivation toward experiencing positive experiences while using technologies increases their affective engagement toward tourism event outcomes [36].

Given that affective engagement in holidays helps predict tourists’ emotions [9], and given that emotions are a post hoc feeling of attributing different event outcomes to different causes [12], the “causality” concept is a stimulator in such a holiday attribution process. This means that with dark technology traits during holidays and tourists’ desires to use technology to facilitate their holidays, DFT service providers/tourists will be confused in predicting such contradictory emotions and causalities concerning engagement in DFT. This is because causality results from individuals’ control over holiday events [35]. Therefore, the feelings of holiday control, desire to use technology, desire to avoid digital technology traits, and navigation of self-emotions all occur in tourists’ mindsets. Thus, we aim to understand and investigate all these assumptions in a better methodological way. 

## 3. Materials and Methods

The study uses semi-structured interviews that allow more inductive logic and flexibility, as participants were required to present answers with fewer constraints [38]. The methodology for conducting the interviews is performed as follows (selection of the interviewees, interview procedures, interview questions, and coding procedures):

### 3.1. Selection of the Interviewees

Professional tour operators who have worked in the tourism field for a long time were responsible for finding voluntary participants to take part in this study. The participants were notified entirely by tour operators about the research aim and objectives to assure transparency when participating and/or choosing the interviewees. The study focused on choosing millennial tourists to participate as volunteer candidates. The reasons behind choosing millennial tourists was that millennials are considered digital natives who grew up with technologies in their private lives. Moreover, millennials were influenced by technologies at a very early stage and developed solid skills concerning digital technologies, which demonstrate an expanded need for regular connectivity [21].

### 3.2. The Interview Procedures

In total, 20 participants (see Table 1) who have experienced DFT holidays were chosen to participate in online interviews via different online platforms, according to the online platforms convenient for participants (e.g., Zoom, Microsoft Teams, and Google Meet). The reason behind choosing online instead of offline interviews is the long-haul distance between the participants and the interviewer. The interviews ranged from 45 min to an hour and a half. The participants’ profiles are listed in Table 1, which summarizes each participant’s gender, age, and DFT holiday type and duration. The participants’ genders were mostly balanced, with 9 females (45%) and 11 males (55%); this was a response to Floros et al.’s [21] call to have a gender balance with participants during examination of DFT. Volunteer participants were mostly millennials. Moreover, the majority of holiday types are cultural activities, outdoor activities, and city landmarks. 

#### 3.2.1. The Interview Questions

The study uses adopted and adapted interview questions, taking into concern the previous DFT studies and LOC in the tourism literature as follows:

(a) A set of questions was adapted from Fong et al. [15] and Jackson [12] to measure tourists’ locus of control when using (vs. not) digital technologies and the Internet during holidays.
  (Do you feel that you almost certainly when you make decisions to engage in DFT? Do you can pretty much determine what will happen when you avoid using digital devices during the journey? Are you able to control your trip itinerary and self-control during the journey without an Internet environment or digital devices? How do you think that you can protect your own personal interests during the holiday without mobile phones?)
(b) A set of questions was adapted from Li et al. [9] to measure the outcome of using (vs. not) digital technologies and the Internet during holidays.
  (Please describe your experience of the digital-free holiday circumstances where you temporarily chose to switch off digital devices, stayed in a disconnected environment (including accommodations) or unexpectedly lost mobile reception and Internet connection? What personal characteristics and individual qualities do you think you need to cope with digital-free situations? and please think of in what ways digital-free tourism experience could help with your self-growth and post-trip well-being?)
(c) A set of questions was adapted from Cai et al. [4] to measure the outcome of using (vs. not) digital technologies and the Internet during holidays.
  (How do you usually use technology while you are traveling? What was your total trip duration? How do you typically use technology while you are traveling? How did this affect the planning of the trip? Note down things you do differently, and how do you feel before, during, and after the departure without using any digital technologies?)
(d) Finally, a set of questions was adopted from Egger et al. [10] to measure the motives of using (vs. not) digital technologies and the Internet during holidays.
  (Why was this trip undertaken (DFT)? What motivated the decision? Do you feel satisfied with the experience after getting rid of digital technology usage?)

These questions help to understand the essence behind participants’ feelings and locus of control while unplugging from any digital technologies during holidays.

#### 3.2.2. The Coding Procedures

All participant comments were transcribed and passed through a thematic and inductive approach because the thematic analysis provides more room and flexibility in interpreting data [39]. Their answers were partially transcribed by “fits” with the aforementioned research scope. This helps crystallize the data about their different LOC brands concerning whether they feel they control their holidays’ events (vs. not). The texts were then read and re-read—as part of the identification process—before the study began on the initial coding [10] to investigate the interviews’ most crucial key theme patterns, especially concerning the repetitiveness and prevalence of participants’ comments for DFT. 

Then, the study implied three provisional parts: DFT benefits as outcomes of tourists’ internal LOC, DFT control difficulties as outcomes of tourists’ external locus of control, and the interdependence between DFT benefits and tourists’ locus of control.

## 4. Results and Discussion

### 4.1. DFT Benefits as an Outcome of Tourists’ Internal Locus of Control

Some participants indicated that their feeling that they could control their DFT holidays helped them perceive several beneficial activities.

“Controlling our feelings without smartphones and the Internet help us appreciate the details of the day and night like sunrise and sunset, birds flying in the sky, sounds of sea waves, feeling nature, and being relaxed without any disturbances help me recover my mental health and avoid any external stressors.”

(Participant #6,7,10,16,18)

“Reducing curiosity about who will call me, social media notifications, and breaking news help me not worry about anything and get more luxury and relaxation without any stress and have self-reflective.”

(Participant #2,4,6,7,19)

“During these days, I have had a lot of subjective experiences which I am sure would not exist if I had my mobile phone and listened to the contradictory opinions on social media.”

(Participant #1,2,6)

“Actually, I feel that I am enjoying the city without using my mobile phone or the Internet.”

(Participant #14,15,18)

“Controlling the moments and Looking at the details of the street and the nature around you in the city is much better to upload your photos constantly and count the number of likes you get, and this helps me enjoy every moment.”

(Participant #4, 11,17,19,20)

“The most interesting thing is listening to the locals, tasting their food, and listening to their life experiences, this happens in case of our ability to get rid of our phones.”

(Participant #4,5,10,11,12,13,20)

The reasons that led participants to feel motivated to continue without Internet access to make social relationships were that interacting with people has advantages concerning well-being when avoiding technology overuse [37]. Moreover, they feel that they could control the holidays as a result of their self-control and ability to enjoy this type of holiday. Thus, they mentioned that staying in peaceful and natural surroundings without any digital technologies or the Internet mindfully motivates them to enjoy well-being, recover from stress, and relieve self-pressure. This occurs because the daily use of technology is associated with a heightened risk of mental health predicaments [40]. Moreover, some participants indicated that controlling their physical and mental abilities to unplug from digital technologies, especially Internet usage during holidays, has many privileges in terms of improving self-reflection, personal growth, and enjoyment of the present moments.

“In the past, I was embarrassed when I was on a trip and just took my phone and didn’t talk to anyone, but now I can socialize with them.”

(Participant #5,7,11,12,14,15,20)

“I feel more confident and have many experiences when talking to them about my point of views on different topics.”

(Participant #2,6,7,11,16,17,19)

“So, if you are immersed in the destination cultures without focusing on digital technologies, you will be able to get better experiences and more satisfaction.”

(Participant #1,2,3,7,13,18,19,20)

These feelings occur because searching for personal growth motivates individuals to engage in challenges when reducing particular habits—here, digital technology elimination—and/or engage in social campaigns for mental health. All these benefits, when avoiding technologies, persuade millennials that DFT will influence their well-being during holidays.

### 4.2. DFT Control Difficulties as an Outcome of Tourists’ External Locus of Control

Some participants indicated that they could not control their feelings and avoid using digital technologies during holidays because of lack of information.

“It is challenging to search for information about the city’s attraction without using mobile phones and navigator applications, which sometimes makes me angry and anxious.”

(Participant #2,6,7,16)

“When I get rid of my mobile phone and start my vacation, I get nervous. Although I was preparing everything, I lose control when I took the first wrong transport.”

(Participant #9,13,17,14)

“The hardest situation I had was when we found that many restaurants are closed, and we couldn’t find the working times of the restaurants in the tourist vouchers.”

(Participant #1,7,11,12,18,20)

“It takes a long time to ask and find restaurants that suit our taste and needs.”

(Participant #1, 4,7,9,11,12,18,20)

“I felt that I had enough self-confidence and control to speak the Russian language, but I realized that I could not communicate or express myself; my mobile phone helped me a lot in these situations.”

(Participant #5,11,12)

Information plays a prominent role while experiencing different events at a holiday destination. That is why participants illustrated that, before a digital detox holiday booking, they search for more holiday details to avoid any failure at the holiday. However, during the digital detox holiday, they sometimes faced obstacles concerning getting lost, transportation schedules, restaurant working times, and translation services in cases of a lack of language knowledge. A lack of information for some participants may lead to missing having control over the events, leading to dissatisfaction along with destructive behavior. 

Moreover, some participants indicated that they were sometimes very frustrated, and they wanted to switch on their mobile phones.

“Anxiety when feeling lonely—at least when you feel isolated from the closest friends who weren’t with you during the holidays.”

(Participant #1,2,6,11,14,17)

“I feel like I forgot my heart and mind with my mobile phone”

(Participant #1,2,6,11,14,20)

These feelings of frustration may occur because the spread of mobile phone applications allows individuals to pick up their phones and track any news and updates, play games, and engage in long chat conversations [16]. So, when individuals cannot control all these features, they are more likely to feel upset about not engaging in such activities.

Moreover, fewer participants highlighted that they had emergency events that lead them to wish to have their mobile phones, such as seeking pharmacies, hospitals, police stations, a public toilet, and foreign exchange centers.

“I started feeling insecure because I heard my money might be stolen, and I don’t know what I should do or where the police stations and any currency exchange rates.”

(Participant #1,4)

“The funniest thing I wanted to go to any toilet, but I felt embarrassed to ask people on the street, so I wished I had my mobile phone to look for the nearest place with restrooms.”

(Participant #2)

“One of my colleagues got sick, and I was scared. I feel like I couldn’t do anything without my mobile phone, I went quickly and asked random people to call the ambulance, and I felt it would be better to have my mobile phone.”

(Participant #13)

“Technology is useful in emergencies.”

(Participant #4, 13, 20)

Emergencies are one of the most unpredictable events that lead individuals to miss control over a moment they wish to control. That is why interviewees hoped that they had their mobile phones; this occurs because, according to Kannan [17], digital technologies are beneficial in urgent events in daily life.

### 4.3. The Interdependence between DFT Benefits and Tourists’ Locus of Control

When disconnection from the Internet and/or any digital technology occurred, participants who could not control digital technology avoidance and Internet use began to control the situation after learning about the benefits of DFT incrementally.

“At the first stage when I got rid of my mobile phone, I could not control myself; I felt anxious and nervous, wished I could pick up any mobile phone and login on to the Internet. Surprisingly, this feeling of lack of control begins to fade constantly, and I began to feel much better and more controllable in my experience.”

(Participant #7,10,11,17,19)

“This occurred because I made relationships with many amazing locals and new friends and discovered new things in myself; it enhanced my self-reflection, changing my moods to better ones.”

(Participant #5,7,10,11,17,19)

Interviewees highlighted that it takes time to respond and control the positive outcome of DFT. This transformation from not controlling then exploiting digital technologies and withdrawing afterward may occur regarding the individual’s LOC antecedents. An individual’s LOC is an outcome of social learning theory; it clarifies that those individuals control events by obtaining different experience benefits [27]. Thus, individuals who benefit from these experiences are more likely to increase their internal LOC [14]. Internal LOC is more likely to be associated with positive outcomes with a high level of satisfaction [12]. 

Some participants highlighted that there passive resistance occurred because of the feeling that there is a need for an external actor (here, mobile phones without forced disconnection).

“I couldn’t control my emotions to connect with my family and colleagues, and I couldn’t stand without watching anything on my mobile phone. I always use my phone even when I shower, so I felt like I would go crazy without my mobile.”

(Participant #1,2,7, 12,20)

“To be honest, sometimes I couldn’t resist and sought to get a phone from my colleagues to check social media.”

(Participant #6,7,10,11,17)

This passive resistance because of external factors confirms the LOC theory pathway that individuals who ascribe events to external causes are more likely to have adverse psychological outcomes [27]. In this vein, these individuals with external LOC are less likely to cope with contradictory life shocks, and have lower savings and satisfaction levels, especially when they cannot control technology adoptions [14]. This, in turn, according to LOC theory, leads to lower mental health [13]. Especially for female tourists because they have quieter internal LOC compared to males males, which motivates them to ascribe any pressure to external causes instead of withstanding it [41] because males have higher self-control than females when clarifying complex events [34].

## 5. Conclusions

Tourists, as digital natives because of rapid technology changes, are influenced by hyper-information and hyper-connection with digital technology features aimed to facilitate their holiday events [11]. However, digital technology may have drawbacks that influence tourists’ psychological attributes, such as anxiety, stress, and fear of missing out on social media [21]. This drives tourists to engage in digital-free tourism to avoid the potential dark traits of digital technology during their holidays [10]. 

DFT has its advantages and disadvantages regarding tourists’ locus of control. Regarding the advantages (tourists with an internal LOC), the study found that DFT leads to more well-being, mental health, and curiosity to socialize during holidays. These advantages of DFT are consistent with the findings of Li et al. [9,11] regarding of DFT benefits during holidays. The study also found that DFT has disadvantages (concerning tourists with an internal locus of control), especially if tourists lack information about services such as transportation schedules and foreign exchange centers, and lack of language knowledge. DFT also has disadvantages when experiencing emergencies. Additionally, female tourists have lower self-control in hard situations than males [34]. Surprisingly, the study found that perceptions of DFT advantages and disadvantages differ according to individuals’ LOC. 

Tourists with an internal LOC tend to be more motivated and optimistic in considering that they can control different event outcomes [12]. This would enable tourists with internal LOC to control their health, mood, and decision making [27] during digital detox at holidays because the internal LOC might serve as an insurance concerning avoiding damaging confusion [14,15]. In contrast, tourists with an external LOC are more likely to be influenced by external causes and cannot withstand DFT holidays. They are less likely to control themself without digital technologies during holidays because they mainly depend on external factors that affect their choices [13]. This is particularly evident among females because they have move of an external LOC compared to males [27]. However, after perceiving the advantages of events, the controlling processes shift from low to high according to the social learning theory [27]. To recapitulate, in the vein of the findings of the study postulation, locus of control is a necessary cognitive support that demonstrates different LOCs in terms of tourist intentions concerning DFT holidays.

### 5.1. Theoretical Contribution

The study responds to the recent calls of Li et al. [9], Egger et al. [10] and Cai et al. [4] that encourage researchers to investigate the psychological reason that stimulates tourists to follow digital-free tourism as a contemporary tendency to eliminate digital technology during holidays. The study responded to these calls by adding a new construct—tourists’ locus of control—to explore the tourist psychological inferences to engage in DFT holidays. 

The study identifies the mechanisms of personality traits that shape the decision to undergo digital detox during vacations based on locus of control. This (a) extends Fong et al.’s [15] findings of tourists’ adoption of technologies and online reservations regarding their locus of control, and (b) contributes to Li et al. [9] and Cai et al.’s [4] literature studies by illuminating that tourists with internal LOC (vs. external), especially male tourists (vs. females), are more likely to engage in digital detox during holidays. The study also contributes to findings concerning how individuals who have internal LOC tend to immerse themselves in nature, which leads to greater well-being in terms of physical and mental health. This contributes to Egger et al.’s study [10] by clarifying the essence behind tourists’ motivations to avoid using digital technologies during vacations. Therefore, the current study has manifold theoretical contributions by answering the tourism literature gap question, clarifying the stimulus that drives tourists to engage (vs. not) in digital detox appeals during holidays.

### 5.2. Managerial Implications

DFT has advantages in terms of increasing tourists’ well-being and satisfaction depending on their self-control during DFT holidays. The study proposes nuanced propositions to help destination managers to encourage tourists to engage in DFT holidays by avoiding obstacles that may cause low self-control during DFT holidays as follows:

On the one hand, to encourage tourists to undergo digital detox during holidays, it is recommended that destination managers list the benefits of digital detox by highlighting tourists’ reviews concerning experiencing digital detox holidays on their websites. It is also recommended that tourism destination managers prioritize a prominent icon on their websites and/or mobile applications during the reservation, notifying tourists about self-growth opportunities provided by evading digital technology use during a journey [9]. On the other hand, to avoid tourists’ lower self-control as an output because of a lack of information and emergencies during DFT holidays, it is recommended that destination managers distribute informative vouchers that include all information about cities’ tourist help centers, transportation schedules, prices, restaurant opening hours, pharmacies, hospitals, public toilet locations, foreign exchange centers, and police stations.

Furthermore, it is recommended that destination managers ensure a prompt service encounter process, especially waiting times, to ensure that tourists participate in digital-free tourism during holidays. For example, destination managers should avoid waiting times while checking in because greater waiting time increases the likelihood of an external LOC [9]. This, in turn, will motivate tourists to pick up their mobile phones and ignore the DFT challenge. Therefore, destination managers should add a new option to their website to facilitate check-in procedures for tourists before tourists’ arrivals. Additionally, there is a high positive correlation between addiction to digital devices, loneliness, and low self-control—external LOC [42]. Therefore, destination managers should suggest to single DFT tourists destinations with entertainment activities at hotels, in contrast with tourists with companions. 

Tourist orientation from tour operators about new tourism trends helps cope with the lingering distress tourists may encounter during holidays. In our study results, travelers who engage in DFT holiday patterns are more likely to mirror low post-traumatic self-confidence with digital detox. Therefore, tour operators (e.g., individual and/or team organizers at different tourism destinations) have to create pre-DFT orientations in an easy way to avoid DFT obstacles. It is recommended that tour operators clarify and explore the predicted sudden and dramatic low self-confidence or low self-control tourists may encounter during a DFT holiday, the emergencies they may encounter, and the solutions to these obstacles. Such a burning urge for “orientation” helps tourists enjoy their DFT holidays through pre-DFT orientation and helps them to find barriers and avoid them, increasing their sense of control and normalcy.

According to Galvin et al. [27], a shift from an external to a primarily internal mentality leads to positive feelings with high satisfaction and control of the outcomes of their events (DFT holidays). Individuals with external LOC are less likely to control DFT holidays; thus, destination managers may propose the #Digital_Detox_Challenge hashtag with incentives. So, if tourists join and achieve success in this challenge, destination managers could upgrade their reservations with discounts. This will raise the tourists’ self-confidence, encouraging individuals to have internal LOC [12].

### 5.3. Limitations and Future Research

The research has several limitations: First, the representative samples focused on millennial tourists because they are more likely to use digital technologies during holidays. Therefore, it is recommended that future research study other generations. Second, the current study lacks an investigation into whether tourists’ health while using mindfulness apps (e.g., Waze, Spotify, Netflix, and even meditation apps) is better than digital access itself. Therefore, future studies should compare how health for tourists may differ in using (and not) specific application combinations that increase their health and enjoyment due to DFT holidays.

## Figures and Tables

**Table 1:** Participants’ profile and DFT holiday information.

Informant No.	Gender	Age	Nationality	Holiday Location	Holiday Type& Activities	Length of Holiday	Length of DFT Experience/per day	Online Interview Platform Type
1	Female	27	British	USA	Outdoor activities	2 months	3	zoom
2	Female	22	Canadian	Egypt	Sea &snorkeling	4 days	1	zoom
3	Male	26	American	UAE	Shopping, business	7 days	1.5	zoom
4	Male	21	British	Vietnam	Sightseeing	10 days	2	google meet
5	Male	31	Vietnamese	Russia	city landmarks	14 days	1	Microsoft teams
6	Female	34	Egyptian	China	Urban tourist, city landmarks	1 month	3–4	zoom
7	Female	23	Vietnamese	China	Urban tourist, city landmarks	1 month	3–4	zoom
8	Male	25	Macedonian	Germany	Cultural activities, outdoor activities	9 days	2	Microsoft teams
9	Female	28	British	USA	urban activities	2 months	1	google meet
10	Female	25	Indian	south Korea	local cuisine, Cultural activities	6 days	3	zoom
11	Female	24	Italian	Russia	Cultural activities, outdoor activities,	5 days	2	Microsoft teams
12	Male	29	Russian	Russia	Cultural activities, outdoor activities	5 days	2	Microsoft teams
13	Female	27	American	Italy	local cuisine, Cultural activities	8 days	3	zoom
14	Male	28	New Zealander	UK	Urban tourist, city landmarks	24 days	2	zoom
15	Male	31	New Zealander	UK	Urban tourist, city landmarks	8 days	1	Microsoft teams
16	Male	22	American	Egypt	Camping, scuba diving	4 days	1	zoom
17	Female	28	Egyptian	UK	Urban tourist, city landmarks	10 days	2	google meet
18	Male	28	Albanian	Russia	outdoor activities	7 days	1.5	zoom
19	Male	30	Egyptian	Saudi Arabia	Urban tourist, city landmarks	21 days	4	zoom
20	Male	26	British	Egypt	Snorkeling, scuba diving	15 days	2	Microsoft teams

## Data Availability

Data available on request due to privacy/ethical restrictions.
